# Tris{2-meth­oxy-6-[(4-methyl­phenyl)­iminiometh­yl]phenolato-κ^2^ *O*,*O*′}tris­(thio­cyanato-κ*N*)cerium(III)

**DOI:** 10.1107/S1600536809016808

**Published:** 2009-05-14

**Authors:** Jian-Feng Liu, Hui-Duo Xian, Guo-Liang Zhao

**Affiliations:** a Zhejiang Key Laboratory for Reactive Chemistry on Solid Surfaces, Institute of Physical Chemistry, Zhejiang Normal University, Jinhua, Zhejiang 321004, People’s Republic of China; b College of Chemistry and Life Science, Zhejiang Normal University, Jinhua 321004, Zhejiang, People’s Republic of China

## Abstract

The asymmetric unit of the title compound, [Ce(NCS)_3_(C_15_H_15_NO_2_)_3_], contains three Schiff base 2-methoxy-6-[(4-methyl­phenyl)iminometh­yl]­phenol (H*L*) ligands and three independent thio­cyanate ions that coordinate the cerium ion *via* their N atoms. The protonated imine N atoms are involved in an intra­molecular hydrogen bond with the respective phenoxide group. The Ce(III) ion exhibits a coordination number of nine.

## Related literature

For background to Schiff bases and their applications, see: Burrows & Bailar (1966 ▶); Leadbeater & Marco (2002 ▶); Quici *et al.* (2004 ▶); Liu *et al.* (2001 ▶). For related structures, see: Li *et al.* (2008 ▶); Xian *et al.* (2008 ▶); Zhao *et al.* (2007 ▶).
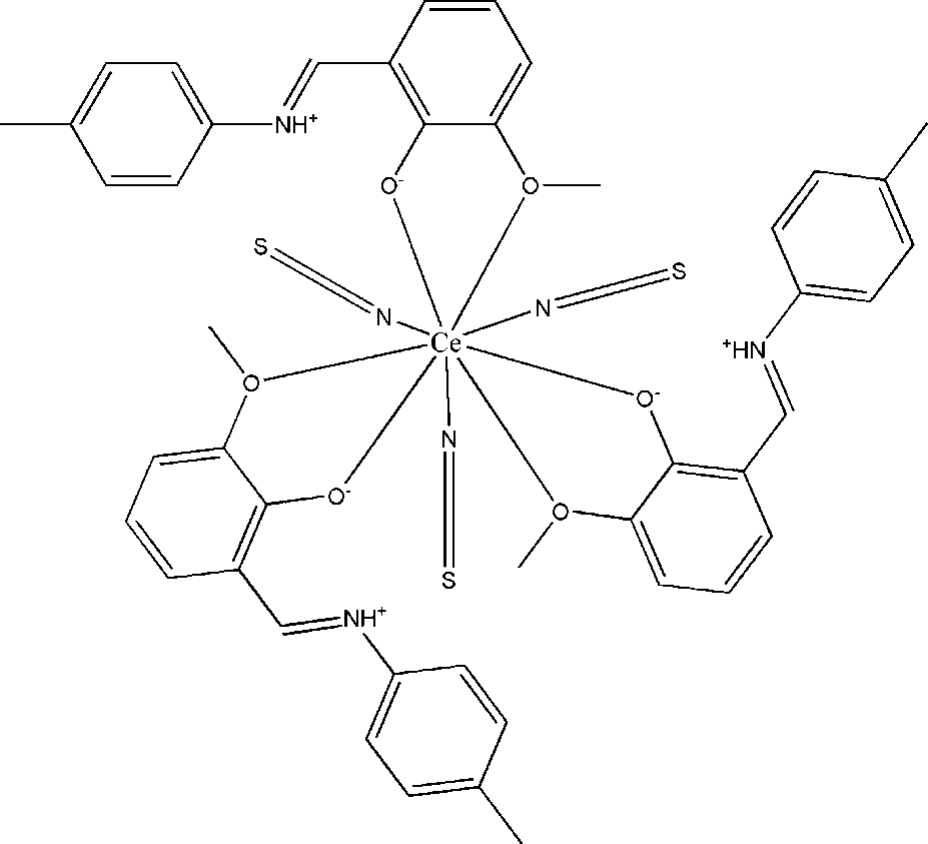


## Experimental

### 

#### Crystal data

[Ce(NCS)_3_(C_15_H_15_NO_2_)_3_]
*M* *_r_* = 1038.23
Monoclinic, 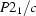

*a* = 16.6730 (3) Å
*b* = 14.2407 (2) Å
*c* = 22.1918 (4) Å
β = 105.979 (1)°
*V* = 5065.53 (15) Å^3^
*Z* = 4
Mo *K*α radiation
μ = 1.07 mm^−1^
*T* = 296 K
0.32 × 0.11 × 0.09 mm

#### Data collection

Bruker APEX2 area-detector diffractometer
Absorption correction: multi-scan (*SADABS*; Sheldrick, 1996 ▶) *T*_min_ = 0.868, *T*_max_ = 0.909
78580 measured reflections
11645 independent reflections
7080 reflections with *I* > 2σ(*I*)
*R*_int_ = 0.093

#### Refinement

*R*[*F* ^2^ > 2σ(*F* ^2^)] = 0.045
*wR*(*F* ^2^) = 0.117
*S* = 1.01
11645 reflections
577 parameters
H-atom parameters constrained
Δρ_max_ = 0.70 e Å^−3^
Δρ_min_ = −0.44 e Å^−3^

### 

Data collection: *APEX2* (Bruker, 2006 ▶); cell refinement: *SAINT* (Bruker, 2006 ▶); data reduction: *SAINT*; program(s) used to solve structure: *SHELXS97* (Sheldrick, 2008 ▶); program(s) used to refine structure: *SHELXL97* (Sheldrick, 2008 ▶); molecular graphics: *SHELXTL* (Sheldrick, 2008 ▶); software used to prepare material for publication: *SHELXTL*.

## Supplementary Material

Crystal structure: contains datablocks I, global. DOI: 10.1107/S1600536809016808/at2767sup1.cif

Structure factors: contains datablocks I. DOI: 10.1107/S1600536809016808/at2767Isup2.hkl

Additional supplementary materials:  crystallographic information; 3D view; checkCIF report

## Figures and Tables

**Table 1:** Hydrogen-bond geometry (Å, °)

*D*—H⋯*A*	*D*—H	H⋯*A*	*D*⋯*A*	*D*—H⋯*A*
N1—H1*A*⋯O1	0.86	1.85	2.556 (4)	138
N2—H2*A*⋯O3	0.86	1.89	2.583 (4)	137
N3—H3*A*⋯O5	0.86	1.88	2.579 (4)	137

**Fig. 1.**
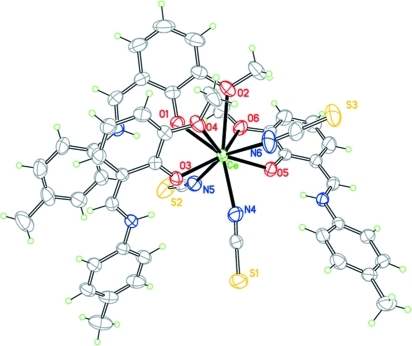
The molecular structure of the title complex, showing the atom-labelling scheme. Displacement ellipsoids are drawn at the 30% probability level.

**Fig. 2.**
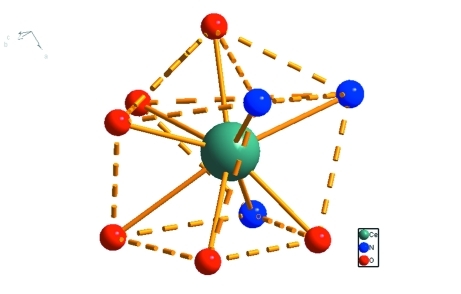
Coordination environment of Ce3+ ion.

## supplementary crystallographic information

### Comment

Schiff base ligands derived from substituted *o*-vanillin and aniline and their rare earth metal complexes have been absorbed considerable attention in the past decades due to their intriguing novel structural features (Burrows & Bailar, 1966; Zhao *et al.*, 2007; Xian *et al.*, 2008; Li *et al.*, 2008) and promising applications in various fields such as catalysis, optoelectronic devices, and so on (Leadbeater & Marco, 2002; Quici *et al.*, 2004). Interested in this field, we have been engaged in a major effort directed toward the development syntheses of new analogous Schiff base derived from *o*-vanillin and their rare metal complexes. In a few of articles we have reported our partial research results (Zhao *et al.*, 2007; Xian *et al.*, 2008; Li *et al.*, 2008). Herein, we describe a new Ce(III) complex, (I).

The single-crystal structure of (I) is shown in Fig.1, which illustrates that the Ce(III) ion in this complex is nine-coordinated by three nitrogen atoms from three thiocyanate ions and six O atoms from the Schiff bases. The Schiff bases coordinated to the Ce(III) ion by didentate mode using O atoms from methoxyl groups and deprotonated phenolic hydroxyl groups. The bonds between Ce(III) and O atoms from phenolic hydroxyl groups are 2.408 (2) Å-2.436 (3) Å, which are longer than the ones between Ce(III) and O atoms of methoxyl groups [2.792 (3) Å-2.838 (3) Å]. And the Ce—N bonds are 2.519 (4)Å -2.576 (4) Å. Because of the geometric and chemical environment requirements of the chelating groups, the coordination geometry deviates considerably from the distorted capped square antiprism geometry (Fig. 2) that gives the lowest energy configuration for nine monodentate ligands surrounding a metal centre (Liu *et al.*, 2001). In one ligand the proton from the phenolic hydroxyl group transfers to imine N atom involving in an intramolecular hydrogen.

### Experimental

Reagents and solvents used were of commercially available quality and without purified before using. The Schiff base ligand 2-[(4- methylphenyl)iminomethyl]-6-methoxy-phenol was sythesized from condensation of *o*-vanillin and *p*-methylaniline. The compound (I) was obtained by adding Ce(NO_3_)_3_ (1 mmol, dissolved in methanol) to *N*-salicylidene-*p*-toluidine)_3_ (3 mmol) in methanol solution. The mixture solution was stirred for 2 h at room temperature. Then 3 mmol NH_4_SCN (dissolved in methanol) was added to the upper solution and the mixture was stirred again for 8 h at room temperature. At last, deposit was filted out and the reddish-brown solution was kept aside. The red crystal was obtained after several days.

### Refinement

The H atoms were positioned geometrically and refined using a riding model [aromatic C—H 0.93 Å, methyl C—H = 0.96 Å, and  *U*_iso_(H) = 1.2 or 1.5*U*_eq_(C)].

### Crystal data

**Table:**

[Ce(NCS)_3_(C_15_H_15_NO_2_)_3_]	*F*(000) = 2116
*M**_r_* = 1038.23	*D*_x_ = 1.361 Mg m^−^^3^
Monoclinic, *P*2_1_/*c*	Mo *K*α radiation, λ = 0.71073 Å
Hall symbol: -P 2ybc	Cell parameters from 5835 reflections
*a* = 16.6730 (3) Å	θ = 1.9–27.5°
*b* = 14.2407 (2) Å	µ = 1.07 mm^−^^1^
*c* = 22.1918 (4) Å	*T* = 296 K
β = 105.979 (1)°	Block, red
*V* = 5065.53 (15) Å^3^	0.32 × 0.11 × 0.09 mm
*Z* = 4	

### Data collection

**Table:**

Bruker APEX2 area-detector diffractometer	11645 independent reflections
Radiation source: fine-focus sealed tube	7080 reflections with *I* > 2σ(*I*)
graphite	*R*_int_ = 0.093
phi and ω scans	θ_max_ = 27.5°, θ_min_ = 1.9°
Absorption correction: multi-scan (*SADABS*; Sheldrick, 1996)	*h* = −21→21
*T*_min_ = 0.868, *T*_max_ = 0.909	*k* = −18→18
78580 measured reflections	*l* = −28→28

### Refinement

**Table:**

Refinement on *F*^2^	Primary atom site location: structure-invariant direct methods
Least-squares matrix: full	Secondary atom site location: difference Fourier map
*R*[*F*^2^ > 2σ(*F*^2^)] = 0.045	Hydrogen site location: inferred from neighbouring sites
*wR*(*F*^2^) = 0.117	H-atom parameters constrained
*S* = 1.01	*w* = 1/[σ^2^(*F*_o_^2^) + (0.0507*P*)^2^ + 0.4997*P*] where *P* = (*F*_o_^2^ + 2*F*_c_^2^)/3
11645 reflections	(Δ/σ)_max_ = 0.001
577 parameters	Δρ_max_ = 0.70 e Å^−^^3^
0 restraints	Δρ_min_ = −0.44 e Å^−^^3^

### Special details

**Table:**

Geometry. All e.s.d.'s (except the e.s.d. in the dihedral angle between two l.s. planes) are estimated using the full covariance matrix. The cell e.s.d.'s are taken into account individually in the estimation of e.s.d.'s in distances, angles and torsion angles; correlations between e.s.d.'s in cell parameters are only used when they are defined by crystal symmetry. An approximate (isotropic) treatment of cell e.s.d.'s is used for estimating e.s.d.'s involving l.s. planes.
Refinement. Refinement of *F*^2^ against ALL reflections. The weighted *R*-factor *wR* and goodness of fit *S* are based on *F*^2^, conventional *R*-factors *R* are based on *F*, with *F* set to zero for negative *F*^2^. The threshold expression of *F*^2^ > σ(*F*^2^) is used only for calculating *R*-factors(gt) *etc*. and is not relevant to the choice of reflections for refinement. *R*-factors based on *F*^2^ are statistically about twice as large as those based on *F*, and *R*- factors based on ALL data will be even larger.

### Fractional atomic coordinates and isotropic or equivalent isotropic displacement parameters (Å^2^)

**Table:**

	*x*	*y*	*z*	*U*_iso_*/*U*_eq_	
Ce	0.288150 (12)	0.224761 (16)	0.107530 (10)	0.04892 (9)	
N1	0.0435 (2)	0.3991 (2)	0.06891 (17)	0.0628 (9)	
H1A	0.0889	0.3757	0.0641	0.075*	
N2	0.01915 (18)	0.1230 (2)	−0.00249 (14)	0.0515 (8)	
H2A	0.0725	0.1260	0.0122	0.062*	
N3	0.52454 (18)	0.0772 (2)	0.08149 (14)	0.0514 (8)	
H3A	0.4756	0.0947	0.0825	0.062*	
N4	0.2818 (2)	0.0638 (3)	0.0534 (2)	0.0775 (11)	
N5	0.2213 (2)	0.2751 (3)	−0.00586 (18)	0.0709 (10)	
N6	0.3894 (3)	0.1422 (4)	0.1973 (2)	0.1028 (16)	
O1	0.19791 (16)	0.35765 (19)	0.11193 (12)	0.0588 (7)	
O2	0.33583 (18)	0.3607 (2)	0.20127 (13)	0.0738 (8)	
O4	0.21751 (17)	0.1993 (2)	0.20688 (12)	0.0667 (8)	
O3	0.14905 (14)	0.16000 (19)	0.08899 (11)	0.0551 (7)	
O5	0.41707 (15)	0.20818 (18)	0.07955 (13)	0.0584 (7)	
O6	0.37866 (18)	0.3824 (2)	0.08337 (14)	0.0704 (8)	
C1	0.1272 (3)	0.4575 (3)	0.1663 (2)	0.0677 (12)	
C2	0.1980 (3)	0.4088 (3)	0.16063 (19)	0.0578 (10)	
C3	0.2725 (3)	0.4159 (3)	0.2102 (2)	0.0682 (12)	
C4	0.2754 (4)	0.4707 (4)	0.2615 (2)	0.0977 (17)	
H4A	0.3248	0.4754	0.2935	0.117*	
C5	0.2048 (5)	0.5196 (5)	0.2658 (3)	0.115 (2)	
H5B	0.2076	0.5570	0.3007	0.138*	
C6	0.1323 (4)	0.5135 (4)	0.2200 (3)	0.0987 (17)	
H6B	0.0856	0.5462	0.2237	0.118*	
C7	0.0516 (3)	0.4487 (3)	0.1193 (2)	0.0709 (13)	
H7A	0.0050	0.4799	0.1245	0.085*	
C8	0.4179 (3)	0.3720 (4)	0.2433 (2)	0.1012 (18)	
H8A	0.4179	0.4237	0.2710	0.152*	
H8B	0.4570	0.3842	0.2195	0.152*	
H8C	0.4337	0.3156	0.2673	0.152*	
C9	−0.0297 (3)	0.3785 (3)	0.0208 (2)	0.0632 (11)	
C10	−0.1078 (3)	0.3844 (4)	0.0299 (3)	0.0831 (14)	
H10A	−0.1139	0.4047	0.0682	0.100*	
C11	−0.1764 (3)	0.3603 (4)	−0.0173 (3)	0.1001 (18)	
H11A	−0.2289	0.3642	−0.0104	0.120*	
C12	−0.1700 (3)	0.3303 (4)	−0.0751 (3)	0.0920 (17)	
C13	−0.0910 (3)	0.3242 (4)	−0.0832 (3)	0.0849 (14)	
H13A	−0.0848	0.3032	−0.1214	0.102*	
C14	−0.0204 (3)	0.3487 (3)	−0.0358 (2)	0.0703 (12)	
H14A	0.0323	0.3451	−0.0423	0.084*	
C15	−0.2448 (4)	0.3010 (5)	−0.1284 (3)	0.139 (3)	
H15A	−0.2950	0.3093	−0.1158	0.208*	
H15B	−0.2393	0.2362	−0.1385	0.208*	
H15C	−0.2473	0.3391	−0.1646	0.208*	
C16	0.5569 (2)	0.2400 (3)	0.0806 (2)	0.0575 (11)	
C17	0.4755 (2)	0.2682 (3)	0.08106 (18)	0.0508 (9)	
C18	0.4593 (3)	0.3654 (3)	0.08333 (19)	0.0592 (10)	
C19	0.5198 (3)	0.4287 (3)	0.0850 (3)	0.0869 (15)	
H19A	0.5079	0.4923	0.0867	0.104*	
C20	0.5990 (3)	0.4020 (4)	0.0841 (3)	0.1019 (19)	
H20A	0.6396	0.4473	0.0853	0.122*	
C21	0.6177 (3)	0.3092 (4)	0.0815 (3)	0.0882 (16)	
H21A	0.6709	0.2914	0.0803	0.106*	
C22	0.5762 (2)	0.1438 (3)	0.0800 (2)	0.0602 (11)	
H22A	0.6296	0.1274	0.0784	0.072*	
C23	0.3494 (3)	0.4778 (3)	0.0765 (3)	0.0939 (17)	
H23A	0.3917	0.5177	0.0686	0.141*	
H23B	0.3369	0.4975	0.1143	0.141*	
H23C	0.2999	0.4818	0.0421	0.141*	
C24	0.5377 (2)	−0.0203 (3)	0.08167 (17)	0.0501 (9)	
C25	0.6082 (3)	−0.0598 (3)	0.07191 (19)	0.0609 (11)	
H25A	0.6500	−0.0217	0.0649	0.073*	
C26	0.6169 (3)	−0.1559 (3)	0.07255 (19)	0.0651 (11)	
H26A	0.6651	−0.1816	0.0659	0.078*	
C27	0.5570 (3)	−0.2153 (3)	0.0826 (2)	0.0644 (11)	
C28	0.4859 (3)	−0.1741 (3)	0.0922 (2)	0.0737 (13)	
H28A	0.4443	−0.2125	0.0992	0.088*	
C29	0.4753 (3)	−0.0776 (3)	0.0916 (2)	0.0649 (11)	
H29A	0.4270	−0.0516	0.0977	0.078*	
C30	0.5667 (3)	−0.3199 (3)	0.0834 (2)	0.0914 (16)	
H30A	0.6192	−0.3358	0.0762	0.137*	
H30B	0.5221	−0.3471	0.0512	0.137*	
H30C	0.5649	−0.3438	0.1235	0.137*	
C31	0.0119 (2)	0.1586 (3)	0.10127 (17)	0.0494 (9)	
C32	0.0991 (2)	0.1697 (3)	0.12478 (18)	0.0490 (9)	
C33	0.1316 (2)	0.1910 (3)	0.18852 (18)	0.0536 (10)	
C34	0.0806 (3)	0.2031 (3)	0.2268 (2)	0.0669 (12)	
H34A	0.1033	0.2179	0.2689	0.080*	
C35	−0.0054 (3)	0.1933 (3)	0.2027 (2)	0.0712 (13)	
H35A	−0.0400	0.2020	0.2287	0.085*	
C36	−0.0391 (3)	0.1712 (3)	0.1416 (2)	0.0666 (12)	
H36A	−0.0966	0.1643	0.1262	0.080*	
C37	−0.0236 (2)	0.1367 (3)	0.03787 (18)	0.0530 (10)	
H37A	−0.0813	0.1315	0.0237	0.064*	
C38	0.2568 (3)	0.2028 (4)	0.2727 (2)	0.0942 (18)	
H38A	0.2162	0.1910	0.2952	0.141*	
H38B	0.2809	0.2638	0.2837	0.141*	
H38C	0.2998	0.1560	0.2836	0.141*	
C39	−0.0113 (2)	0.1037 (3)	−0.06771 (18)	0.0519 (9)	
C40	0.0419 (3)	0.1132 (3)	−0.1039 (2)	0.0717 (12)	
H40A	0.0974	0.1292	−0.0855	0.086*	
C41	0.0142 (4)	0.0993 (4)	−0.1678 (2)	0.0879 (16)	
H41A	0.0513	0.1056	−0.1921	0.106*	
C42	−0.0673 (4)	0.0762 (3)	−0.1959 (2)	0.0792 (14)	
C43	−0.1204 (3)	0.0658 (3)	−0.1590 (2)	0.0753 (13)	
H43A	−0.1756	0.0488	−0.1774	0.090*	
C44	−0.0934 (3)	0.0801 (3)	−0.0949 (2)	0.0639 (11)	
H44A	−0.1303	0.0737	−0.0705	0.077*	
C45	−0.0990 (4)	0.0618 (5)	−0.2663 (2)	0.120 (2)	
H45A	−0.0542	0.0716	−0.2850	0.180*	
H45B	−0.1431	0.1057	−0.2835	0.180*	
H45C	−0.1199	−0.0011	−0.2748	0.180*	
C46	0.2665 (2)	−0.0051 (3)	0.0259 (2)	0.0626 (11)	
C47	0.1987 (3)	0.3255 (3)	−0.0465 (2)	0.0617 (11)	
C48	0.4580 (4)	0.1386 (4)	0.2277 (2)	0.0909 (16)	
S1	0.24756 (8)	−0.10483 (11)	−0.01078 (8)	0.1025 (5)	
S2	0.16650 (12)	0.39843 (13)	−0.10448 (7)	0.1192 (6)	
S3	0.55281 (11)	0.13689 (15)	0.27002 (10)	0.1500 (8)	

### Atomic displacement parameters (Å^2^)

**Table:**

	*U*^11^	*U*^22^	*U*^33^	*U*^12^	*U*^13^	*U*^23^
Ce	0.04068 (12)	0.05215 (15)	0.04987 (15)	0.00344 (10)	0.00563 (9)	0.00376 (12)
N1	0.055 (2)	0.059 (2)	0.071 (2)	0.0126 (17)	0.0131 (19)	0.006 (2)
N2	0.0464 (17)	0.056 (2)	0.0494 (19)	−0.0025 (15)	0.0082 (15)	−0.0033 (16)
N3	0.0471 (17)	0.048 (2)	0.058 (2)	0.0034 (15)	0.0128 (15)	0.0025 (16)
N4	0.063 (2)	0.068 (3)	0.106 (3)	−0.005 (2)	0.030 (2)	−0.016 (2)
N5	0.073 (2)	0.081 (3)	0.055 (2)	0.010 (2)	0.011 (2)	0.006 (2)
N6	0.076 (3)	0.142 (5)	0.079 (3)	0.033 (3)	0.004 (2)	0.038 (3)
O1	0.0658 (17)	0.0602 (18)	0.0464 (16)	0.0091 (13)	0.0089 (13)	−0.0029 (14)
O2	0.0677 (19)	0.087 (2)	0.0532 (18)	−0.0037 (17)	−0.0063 (15)	0.0047 (16)
O4	0.0588 (17)	0.092 (2)	0.0427 (16)	−0.0094 (15)	0.0025 (13)	0.0086 (15)
O3	0.0458 (14)	0.0679 (19)	0.0523 (16)	−0.0030 (13)	0.0146 (12)	−0.0054 (14)
O5	0.0473 (15)	0.0460 (17)	0.083 (2)	−0.0026 (12)	0.0200 (14)	0.0005 (14)
O6	0.0742 (19)	0.0475 (18)	0.090 (2)	0.0081 (14)	0.0242 (17)	0.0017 (16)
C1	0.080 (3)	0.062 (3)	0.064 (3)	0.006 (2)	0.025 (3)	−0.008 (2)
C2	0.075 (3)	0.051 (3)	0.047 (2)	0.000 (2)	0.016 (2)	0.002 (2)
C3	0.080 (3)	0.067 (3)	0.053 (3)	−0.005 (3)	0.010 (2)	−0.001 (2)
C4	0.113 (4)	0.116 (5)	0.058 (3)	−0.023 (4)	0.014 (3)	−0.025 (3)
C5	0.140 (6)	0.128 (6)	0.081 (4)	−0.017 (5)	0.037 (4)	−0.051 (4)
C6	0.119 (5)	0.095 (4)	0.090 (4)	0.010 (4)	0.041 (4)	−0.029 (3)
C7	0.079 (3)	0.056 (3)	0.084 (3)	0.013 (2)	0.033 (3)	−0.002 (3)
C8	0.079 (3)	0.104 (4)	0.093 (4)	−0.015 (3)	−0.021 (3)	0.003 (3)
C9	0.062 (3)	0.048 (3)	0.073 (3)	0.015 (2)	0.009 (2)	0.006 (2)
C10	0.064 (3)	0.080 (4)	0.103 (4)	0.023 (3)	0.019 (3)	0.002 (3)
C11	0.064 (3)	0.096 (4)	0.133 (5)	0.022 (3)	0.015 (4)	0.008 (4)
C12	0.062 (3)	0.069 (4)	0.123 (5)	0.013 (3)	−0.011 (3)	0.024 (3)
C13	0.090 (4)	0.061 (3)	0.090 (4)	0.009 (3)	0.001 (3)	0.004 (3)
C14	0.066 (3)	0.056 (3)	0.083 (3)	0.007 (2)	0.010 (3)	0.009 (3)
C15	0.093 (4)	0.114 (5)	0.163 (6)	0.008 (4)	−0.043 (4)	0.001 (5)
C16	0.050 (2)	0.052 (3)	0.072 (3)	−0.0047 (18)	0.019 (2)	0.000 (2)
C17	0.055 (2)	0.044 (2)	0.052 (2)	−0.0066 (19)	0.0131 (19)	−0.0009 (19)
C18	0.064 (3)	0.049 (3)	0.065 (3)	0.001 (2)	0.018 (2)	−0.001 (2)
C19	0.088 (4)	0.050 (3)	0.125 (5)	−0.012 (3)	0.034 (3)	−0.004 (3)
C20	0.084 (4)	0.063 (4)	0.165 (6)	−0.031 (3)	0.046 (4)	−0.014 (4)
C21	0.062 (3)	0.071 (3)	0.136 (5)	−0.012 (3)	0.035 (3)	−0.005 (3)
C22	0.050 (2)	0.056 (3)	0.075 (3)	0.001 (2)	0.019 (2)	−0.004 (2)
C23	0.113 (4)	0.045 (3)	0.131 (5)	0.024 (3)	0.046 (4)	0.017 (3)
C24	0.050 (2)	0.050 (2)	0.045 (2)	0.0018 (18)	0.0046 (18)	0.0014 (19)
C25	0.066 (3)	0.055 (3)	0.064 (3)	−0.002 (2)	0.022 (2)	0.001 (2)
C26	0.076 (3)	0.062 (3)	0.055 (3)	0.014 (2)	0.013 (2)	−0.003 (2)
C27	0.083 (3)	0.047 (3)	0.051 (3)	0.004 (2)	−0.001 (2)	0.000 (2)
C28	0.072 (3)	0.059 (3)	0.080 (3)	−0.013 (2)	0.004 (3)	0.006 (3)
C29	0.054 (2)	0.061 (3)	0.073 (3)	−0.001 (2)	0.006 (2)	0.002 (2)
C30	0.122 (4)	0.052 (3)	0.087 (4)	0.000 (3)	0.006 (3)	−0.007 (3)
C31	0.047 (2)	0.053 (2)	0.048 (2)	−0.0025 (17)	0.0124 (18)	−0.0020 (19)
C32	0.050 (2)	0.049 (2)	0.049 (2)	−0.0006 (18)	0.0152 (18)	0.0036 (19)
C33	0.057 (2)	0.051 (2)	0.051 (2)	−0.0061 (19)	0.013 (2)	0.0070 (19)
C34	0.079 (3)	0.073 (3)	0.049 (2)	−0.004 (2)	0.018 (2)	−0.001 (2)
C35	0.072 (3)	0.084 (3)	0.068 (3)	−0.008 (2)	0.036 (3)	−0.009 (3)
C36	0.053 (2)	0.083 (3)	0.068 (3)	−0.002 (2)	0.024 (2)	−0.002 (3)
C37	0.047 (2)	0.053 (2)	0.058 (3)	0.0000 (18)	0.013 (2)	0.002 (2)
C38	0.077 (3)	0.153 (6)	0.040 (3)	−0.018 (3)	−0.005 (2)	0.014 (3)
C39	0.057 (2)	0.049 (2)	0.047 (2)	0.0000 (18)	0.0089 (19)	−0.0025 (19)
C40	0.069 (3)	0.081 (3)	0.067 (3)	−0.015 (2)	0.022 (2)	−0.015 (3)
C41	0.115 (4)	0.092 (4)	0.065 (3)	−0.026 (3)	0.040 (3)	−0.019 (3)
C42	0.114 (4)	0.066 (3)	0.054 (3)	−0.013 (3)	0.018 (3)	−0.009 (2)
C43	0.074 (3)	0.075 (3)	0.064 (3)	−0.010 (2)	−0.002 (3)	−0.012 (3)
C44	0.060 (3)	0.068 (3)	0.061 (3)	−0.005 (2)	0.013 (2)	−0.006 (2)
C45	0.176 (6)	0.114 (5)	0.059 (3)	−0.029 (4)	0.014 (4)	−0.020 (3)
C46	0.043 (2)	0.068 (3)	0.080 (3)	−0.011 (2)	0.021 (2)	−0.012 (3)
C47	0.070 (3)	0.072 (3)	0.043 (2)	0.017 (2)	0.015 (2)	−0.008 (2)
C48	0.092 (4)	0.094 (4)	0.077 (4)	0.035 (3)	0.008 (3)	0.017 (3)
S1	0.0706 (8)	0.0955 (10)	0.1541 (14)	−0.0343 (7)	0.0519 (9)	−0.0574 (10)
S2	0.1679 (15)	0.1299 (14)	0.0722 (9)	0.0734 (12)	0.0540 (10)	0.0403 (9)
S3	0.0956 (12)	0.1407 (17)	0.1694 (18)	0.0401 (11)	−0.0382 (12)	−0.0171 (14)

### Geometric parameters (Å, °)

**Table:**

Ce—O5	2.408 (2)	C16—C21	1.410 (6)
Ce—O3	2.423 (2)	C16—C17	1.418 (5)
Ce—O1	2.436 (3)	C17—C18	1.414 (5)
Ce—N6	2.519 (4)	C18—C19	1.346 (6)
Ce—N5	2.558 (4)	C19—C20	1.379 (7)
Ce—N4	2.576 (4)	C19—H19A	0.9300
Ce—O2	2.792 (3)	C20—C21	1.363 (7)
Ce—O4	2.795 (3)	C20—H20A	0.9300
Ce—O6	2.838 (3)	C21—H21A	0.9300
N1—C7	1.297 (5)	C22—H22A	0.9300
N1—C9	1.414 (5)	C23—H23A	0.9600
N1—H1A	0.8600	C23—H23B	0.9600
N2—C37	1.303 (4)	C23—H23C	0.9600
N2—C39	1.422 (5)	C24—C25	1.373 (5)
N2—H2A	0.8600	C24—C29	1.386 (5)
N3—C22	1.288 (5)	C25—C26	1.376 (6)
N3—C24	1.406 (5)	C25—H25A	0.9300
N3—H3A	0.8600	C26—C27	1.373 (6)
N4—C46	1.148 (5)	C26—H26A	0.9300
N5—C47	1.132 (5)	C27—C28	1.391 (6)
N6—C48	1.158 (6)	C27—C30	1.498 (6)
O1—C2	1.303 (4)	C28—C29	1.385 (6)
O2—C3	1.374 (5)	C28—H28A	0.9300
O2—C8	1.437 (5)	C29—H29A	0.9300
O4—C33	1.383 (4)	C30—H30A	0.9600
O4—C38	1.429 (5)	C30—H30B	0.9600
O3—C32	1.307 (4)	C30—H30C	0.9600
O5—C17	1.289 (4)	C31—C37	1.403 (5)
O6—C18	1.367 (5)	C31—C36	1.406 (5)
O6—C23	1.436 (5)	C31—C32	1.411 (5)
C1—C7	1.403 (6)	C32—C33	1.402 (5)
C1—C2	1.405 (6)	C33—C34	1.368 (5)
C1—C6	1.417 (6)	C34—C35	1.393 (6)
C2—C3	1.418 (6)	C34—H34A	0.9300
C3—C4	1.369 (6)	C35—C36	1.354 (6)
C4—C5	1.394 (8)	C35—H35A	0.9300
C4—H4A	0.9300	C36—H36A	0.9300
C5—C6	1.351 (8)	C37—H37A	0.9300
C5—H5B	0.9300	C38—H38A	0.9600
C6—H6B	0.9300	C38—H38B	0.9600
C7—H7A	0.9300	C38—H38C	0.9600
C8—H8A	0.9600	C39—C40	1.358 (5)
C8—H8B	0.9600	C39—C44	1.377 (5)
C8—H8C	0.9600	C40—C41	1.379 (6)
C9—C14	1.375 (6)	C40—H40A	0.9300
C9—C10	1.374 (6)	C41—C42	1.370 (7)
C10—C11	1.364 (7)	C41—H41A	0.9300
C10—H10A	0.9300	C42—C43	1.368 (6)
C11—C12	1.385 (8)	C42—C45	1.519 (6)
C11—H11A	0.9300	C43—C44	1.384 (6)
C12—C13	1.380 (7)	C43—H43A	0.9300
C12—C15	1.522 (8)	C44—H44A	0.9300
C13—C14	1.390 (6)	C45—H45A	0.9600
C13—H13A	0.9300	C45—H45B	0.9600
C14—H14A	0.9300	C45—H45C	0.9600
C15—H15A	0.9600	C46—S1	1.623 (5)
C15—H15B	0.9600	C47—S2	1.626 (5)
C15—H15C	0.9600	C48—S3	1.601 (6)
C16—C22	1.409 (5)		
			
O5—Ce—O3	143.08 (9)	C12—C15—H15C	109.5
O5—Ce—O1	133.75 (9)	H15A—C15—H15C	109.5
O3—Ce—O1	74.32 (9)	H15B—C15—H15C	109.5
O5—Ce—N6	72.93 (12)	C22—C16—C21	120.9 (4)
O3—Ce—N6	111.00 (13)	C22—C16—C17	119.9 (3)
O1—Ce—N6	128.14 (12)	C21—C16—C17	119.2 (4)
O5—Ce—N5	87.26 (11)	O5—C17—C18	120.0 (3)
O3—Ce—N5	78.58 (11)	O5—C17—C16	122.0 (4)
O1—Ce—N5	73.43 (10)	C18—C17—C16	118.0 (4)
N6—Ce—N5	157.54 (13)	C19—C18—O6	127.7 (4)
O5—Ce—N4	73.60 (10)	C19—C18—C17	120.6 (4)
O3—Ce—N4	70.59 (10)	O6—C18—C17	111.8 (3)
O1—Ce—N4	139.75 (10)	C18—C19—C20	121.9 (5)
N6—Ce—N4	83.45 (15)	C18—C19—H19A	119.1
N5—Ce—N4	80.81 (13)	C20—C19—H19A	119.1
O5—Ce—O2	99.72 (9)	C21—C20—C19	119.9 (4)
O3—Ce—O2	116.95 (8)	C21—C20—H20A	120.0
O1—Ce—O2	58.93 (9)	C19—C20—H20A	120.0
N6—Ce—O2	75.11 (14)	C20—C21—C16	120.4 (4)
N5—Ce—O2	119.74 (11)	C20—C21—H21A	119.8
N4—Ce—O2	158.56 (11)	C16—C21—H21A	119.8
O5—Ce—O4	142.27 (9)	N3—C22—C16	124.0 (4)
O3—Ce—O4	59.54 (8)	N3—C22—H22A	118.0
O1—Ce—O4	70.74 (9)	C16—C22—H22A	118.0
N6—Ce—O4	69.64 (11)	O6—C23—H23A	109.5
N5—Ce—O4	130.40 (10)	O6—C23—H23B	109.5
N4—Ce—O4	106.26 (10)	H23A—C23—H23B	109.5
O2—Ce—O4	66.27 (9)	O6—C23—H23C	109.5
O5—Ce—O6	57.99 (8)	H23A—C23—H23C	109.5
O3—Ce—O6	143.41 (9)	H23B—C23—H23C	109.5
O1—Ce—O6	76.07 (9)	C25—C24—C29	119.8 (4)
N6—Ce—O6	104.06 (13)	C25—C24—N3	122.9 (3)
N5—Ce—O6	72.71 (11)	C29—C24—N3	117.3 (3)
N4—Ce—O6	124.87 (10)	C24—C25—C26	119.8 (4)
O2—Ce—O6	62.29 (8)	C24—C25—H25A	120.1
O4—Ce—O6	127.83 (8)	C26—C25—H25A	120.1
C7—N1—C9	128.8 (4)	C27—C26—C25	122.4 (4)
C7—N1—H1A	115.6	C27—C26—H26A	118.8
C9—N1—H1A	115.6	C25—C26—H26A	118.8
C37—N2—C39	128.2 (3)	C26—C27—C28	117.0 (4)
C37—N2—H2A	115.9	C26—C27—C30	122.3 (4)
C39—N2—H2A	115.9	C28—C27—C30	120.7 (4)
C22—N3—C24	128.5 (3)	C29—C28—C27	122.0 (4)
C22—N3—H3A	115.7	C29—C28—H28A	119.0
C24—N3—H3A	115.7	C27—C28—H28A	119.0
C46—N4—Ce	169.8 (3)	C28—C29—C24	119.1 (4)
C47—N5—Ce	157.0 (4)	C28—C29—H29A	120.5
C48—N6—Ce	145.1 (5)	C24—C29—H29A	120.5
C2—O1—Ce	127.4 (2)	C27—C30—H30A	109.5
C3—O2—C8	118.3 (4)	C27—C30—H30B	109.5
C3—O2—Ce	115.6 (2)	H30A—C30—H30B	109.5
C8—O2—Ce	125.8 (3)	C27—C30—H30C	109.5
C33—O4—C38	116.8 (3)	H30A—C30—H30C	109.5
C33—O4—Ce	114.0 (2)	H30B—C30—H30C	109.5
C38—O4—Ce	128.9 (2)	C37—C31—C36	120.3 (3)
C32—O3—Ce	126.7 (2)	C37—C31—C32	120.2 (3)
C17—O5—Ce	130.5 (2)	C36—C31—C32	119.5 (4)
C18—O6—C23	118.3 (3)	O3—C32—C33	120.1 (3)
C18—O6—Ce	115.7 (2)	O3—C32—C31	121.8 (3)
C23—O6—Ce	125.8 (3)	C33—C32—C31	118.1 (3)
C7—C1—C2	119.6 (4)	C34—C33—O4	125.2 (4)
C7—C1—C6	120.7 (5)	C34—C33—C32	121.4 (4)
C2—C1—C6	119.7 (5)	O4—C33—C32	113.4 (3)
O1—C2—C1	122.8 (4)	C33—C34—C35	119.8 (4)
O1—C2—C3	119.0 (4)	C33—C34—H34A	120.1
C1—C2—C3	118.3 (4)	C35—C34—H34A	120.1
C4—C3—O2	126.4 (5)	C36—C35—C34	120.6 (4)
C4—C3—C2	120.6 (5)	C36—C35—H35A	119.7
O2—C3—C2	113.0 (4)	C34—C35—H35A	119.7
C3—C4—C5	120.3 (5)	C35—C36—C31	120.6 (4)
C3—C4—H4A	119.8	C35—C36—H36A	119.7
C5—C4—H4A	119.8	C31—C36—H36A	119.7
C6—C5—C4	120.9 (5)	N2—C37—C31	124.3 (3)
C6—C5—H5B	119.6	N2—C37—H37A	117.9
C4—C5—H5B	119.6	C31—C37—H37A	117.9
C5—C6—C1	120.3 (5)	O4—C38—H38A	109.5
C5—C6—H6B	119.9	O4—C38—H38B	109.5
C1—C6—H6B	119.9	H38A—C38—H38B	109.5
N1—C7—C1	123.3 (4)	O4—C38—H38C	109.5
N1—C7—H7A	118.4	H38A—C38—H38C	109.5
C1—C7—H7A	118.4	H38B—C38—H38C	109.5
O2—C8—H8A	109.5	C40—C39—C44	119.8 (4)
O2—C8—H8B	109.5	C40—C39—N2	118.3 (4)
H8A—C8—H8B	109.5	C44—C39—N2	121.8 (3)
O2—C8—H8C	109.5	C39—C40—C41	120.3 (4)
H8A—C8—H8C	109.5	C39—C40—H40A	119.8
H8B—C8—H8C	109.5	C41—C40—H40A	119.8
C14—C9—C10	120.2 (5)	C42—C41—C40	120.8 (4)
C14—C9—N1	117.5 (4)	C42—C41—H41A	119.6
C10—C9—N1	122.2 (4)	C40—C41—H41A	119.6
C11—C10—C9	120.0 (5)	C43—C42—C41	118.5 (4)
C11—C10—H10A	120.0	C43—C42—C45	120.0 (5)
C9—C10—H10A	120.0	C41—C42—C45	121.4 (5)
C10—C11—C12	121.7 (5)	C42—C43—C44	121.2 (4)
C10—C11—H11A	119.1	C42—C43—H43A	119.4
C12—C11—H11A	119.1	C44—C43—H43A	119.4
C13—C12—C11	117.4 (5)	C39—C44—C43	119.3 (4)
C13—C12—C15	119.3 (6)	C39—C44—H44A	120.4
C11—C12—C15	123.3 (6)	C43—C44—H44A	120.4
C12—C13—C14	121.7 (5)	C42—C45—H45A	109.5
C12—C13—H13A	119.2	C42—C45—H45B	109.5
C14—C13—H13A	119.2	H45A—C45—H45B	109.5
C9—C14—C13	118.9 (4)	C42—C45—H45C	109.5
C9—C14—H14A	120.5	H45A—C45—H45C	109.5
C13—C14—H14A	120.5	H45B—C45—H45C	109.5
C12—C15—H15A	109.5	N4—C46—S1	177.7 (5)
C12—C15—H15B	109.5	N5—C47—S2	179.5 (4)
H15A—C15—H15B	109.5	N6—C48—S3	178.3 (6)
			
O5—Ce—N4—C46	−145 (2)	C8—O2—C3—C4	−11.6 (7)
O3—Ce—N4—C46	26 (2)	Ce—O2—C3—C4	163.4 (4)
O1—Ce—N4—C46	−5(2)	C8—O2—C3—C2	170.8 (4)
N6—Ce—N4—C46	141 (2)	Ce—O2—C3—C2	−14.2 (4)
N5—Ce—N4—C46	−55 (2)	O1—C2—C3—C4	178.5 (4)
O2—Ce—N4—C46	141 (2)	C1—C2—C3—C4	−1.6 (7)
O4—Ce—N4—C46	74 (2)	O1—C2—C3—O2	−3.8 (6)
O6—Ce—N4—C46	−117 (2)	C1—C2—C3—O2	176.1 (4)
O5—Ce—N5—C47	−93.6 (9)	O2—C3—C4—C5	−176.7 (5)
O3—Ce—N5—C47	120.7 (9)	C2—C3—C4—C5	0.7 (8)
O1—Ce—N5—C47	43.8 (9)	C3—C4—C5—C6	0.4 (10)
N6—Ce—N5—C47	−121.4 (9)	C4—C5—C6—C1	−0.5 (10)
N4—Ce—N5—C47	−167.4 (9)	C7—C1—C6—C5	178.3 (5)
O2—Ce—N5—C47	6.0 (9)	C2—C1—C6—C5	−0.5 (8)
O4—Ce—N5—C47	89.0 (9)	C9—N1—C7—C1	174.4 (4)
O6—Ce—N5—C47	−36.4 (9)	C2—C1—C7—N1	−1.9 (7)
O5—Ce—N6—C48	39.4 (8)	C6—C1—C7—N1	179.3 (4)
O3—Ce—N6—C48	−179.5 (8)	C7—N1—C9—C14	161.3 (4)
O1—Ce—N6—C48	−93.3 (8)	C7—N1—C9—C10	−20.9 (7)
N5—Ce—N6—C48	68.6 (10)	C14—C9—C10—C11	0.2 (7)
N4—Ce—N6—C48	114.2 (8)	N1—C9—C10—C11	−177.5 (5)
O2—Ce—N6—C48	−65.9 (8)	C9—C10—C11—C12	−0.4 (8)
O4—Ce—N6—C48	−135.7 (8)	C10—C11—C12—C13	0.9 (8)
O6—Ce—N6—C48	−10.1 (8)	C10—C11—C12—C15	178.8 (5)
O5—Ce—O1—C2	−95.1 (3)	C11—C12—C13—C14	−1.1 (8)
O3—Ce—O1—C2	113.1 (3)	C15—C12—C13—C14	−179.1 (5)
N6—Ce—O1—C2	8.5 (4)	C10—C9—C14—C13	−0.4 (7)
N5—Ce—O1—C2	−164.4 (3)	N1—C9—C14—C13	177.4 (4)
N4—Ce—O1—C2	143.2 (3)	C12—C13—C14—C9	0.9 (7)
O2—Ce—O1—C2	−22.8 (3)	Ce—O5—C17—C18	−18.9 (6)
O4—Ce—O1—C2	50.5 (3)	Ce—O5—C17—C16	161.0 (3)
O6—Ce—O1—C2	−88.6 (3)	C22—C16—C17—O5	−1.5 (6)
O5—Ce—O2—C3	153.7 (3)	C21—C16—C17—O5	179.1 (4)
O3—Ce—O2—C3	−30.7 (3)	C22—C16—C17—C18	178.4 (4)
O1—Ce—O2—C3	18.0 (3)	C21—C16—C17—C18	−1.0 (6)
N6—Ce—O2—C3	−137.0 (3)	C23—O6—C18—C19	8.5 (7)
N5—Ce—O2—C3	61.3 (3)	Ce—O6—C18—C19	−166.5 (4)
N4—Ce—O2—C3	−136.7 (3)	C23—O6—C18—C17	−171.3 (4)
O4—Ce—O2—C3	−63.1 (3)	Ce—O6—C18—C17	13.7 (4)
O6—Ce—O2—C3	107.9 (3)	O5—C17—C18—C19	180.0 (4)
O5—Ce—O2—C8	−31.8 (4)	C16—C17—C18—C19	0.1 (6)
O3—Ce—O2—C8	143.8 (3)	O5—C17—C18—O6	−0.2 (6)
O1—Ce—O2—C8	−167.5 (4)	C16—C17—C18—O6	179.9 (4)
N6—Ce—O2—C8	37.5 (3)	O6—C18—C19—C20	−179.4 (5)
N5—Ce—O2—C8	−124.2 (3)	C17—C18—C19—C20	0.4 (8)
N4—Ce—O2—C8	37.9 (5)	C18—C19—C20—C21	0.0 (9)
O4—Ce—O2—C8	111.5 (4)	C19—C20—C21—C16	−1.0 (9)
O6—Ce—O2—C8	−77.5 (3)	C22—C16—C21—C20	−177.9 (5)
O5—Ce—O4—C33	−158.6 (2)	C17—C16—C21—C20	1.4 (8)
O3—Ce—O4—C33	−19.6 (2)	C24—N3—C22—C16	−179.3 (4)
O1—Ce—O4—C33	63.2 (3)	C21—C16—C22—N3	177.4 (4)
N6—Ce—O4—C33	−151.0 (3)	C17—C16—C22—N3	−2.0 (7)
N5—Ce—O4—C33	17.1 (3)	C22—N3—C24—C25	−8.8 (6)
N4—Ce—O4—C33	−74.6 (3)	C22—N3—C24—C29	171.9 (4)
O2—Ce—O4—C33	126.8 (3)	C29—C24—C25—C26	−0.6 (6)
O6—Ce—O4—C33	116.7 (3)	N3—C24—C25—C26	−179.8 (4)
O5—Ce—O4—C38	28.0 (4)	C24—C25—C26—C27	0.1 (7)
O3—Ce—O4—C38	167.1 (4)	C25—C26—C27—C28	0.1 (6)
O1—Ce—O4—C38	−110.2 (4)	C25—C26—C27—C30	−179.8 (4)
N6—Ce—O4—C38	35.7 (4)	C26—C27—C28—C29	0.2 (7)
N5—Ce—O4—C38	−156.2 (4)	C30—C27—C28—C29	−179.9 (4)
N4—Ce—O4—C38	112.1 (4)	C27—C28—C29—C24	−0.6 (7)
O2—Ce—O4—C38	−46.5 (4)	C25—C24—C29—C28	0.8 (6)
O6—Ce—O4—C38	−56.6 (4)	N3—C24—C29—C28	−179.9 (4)
O5—Ce—O3—C32	160.2 (3)	Ce—O3—C32—C33	−22.8 (5)
O1—Ce—O3—C32	−54.5 (3)	Ce—O3—C32—C31	157.6 (3)
N6—Ce—O3—C32	70.9 (3)	C37—C31—C32—O3	0.0 (6)
N5—Ce—O3—C32	−130.3 (3)	C36—C31—C32—O3	−179.0 (4)
N4—Ce—O3—C32	145.6 (3)	C37—C31—C32—C33	−179.6 (4)
O2—Ce—O3—C32	−12.5 (3)	C36—C31—C32—C33	1.3 (6)
O4—Ce—O3—C32	22.1 (3)	C38—O4—C33—C34	12.6 (6)
O6—Ce—O3—C32	−91.6 (3)	Ce—O4—C33—C34	−161.6 (3)
O3—Ce—O5—C17	156.3 (3)	C38—O4—C33—C32	−168.2 (4)
O1—Ce—O5—C17	25.6 (4)	Ce—O4—C33—C32	17.6 (4)
N6—Ce—O5—C17	−101.3 (4)	O3—C32—C33—C34	178.9 (4)
N5—Ce—O5—C17	89.4 (3)	C31—C32—C33—C34	−1.5 (6)
N4—Ce—O5—C17	170.7 (4)	O3—C32—C33—O4	−0.3 (5)
O2—Ce—O5—C17	−30.3 (3)	C31—C32—C33—O4	179.3 (3)
O4—Ce—O5—C17	−93.8 (3)	O4—C33—C34—C35	179.7 (4)
O6—Ce—O5—C17	18.2 (3)	C32—C33—C34—C35	0.6 (7)
O5—Ce—O6—C18	−15.5 (3)	C33—C34—C35—C36	0.5 (7)
O3—Ce—O6—C18	−153.3 (2)	C34—C35—C36—C31	−0.6 (7)
O1—Ce—O6—C18	170.0 (3)	C37—C31—C36—C35	−179.4 (4)
N6—Ce—O6—C18	43.6 (3)	C32—C31—C36—C35	−0.3 (7)
N5—Ce—O6—C18	−113.3 (3)	C39—N2—C37—C31	−177.6 (4)
N4—Ce—O6—C18	−48.2 (3)	C36—C31—C37—N2	−179.3 (4)
O2—Ce—O6—C18	108.0 (3)	C32—C31—C37—N2	1.6 (6)
O4—Ce—O6—C18	118.5 (3)	C37—N2—C39—C40	165.0 (4)
O5—Ce—O6—C23	169.9 (4)	C37—N2—C39—C44	−12.1 (6)
O3—Ce—O6—C23	32.2 (4)	C44—C39—C40—C41	0.0 (7)
O1—Ce—O6—C23	−4.6 (3)	N2—C39—C40—C41	−177.1 (4)
N6—Ce—O6—C23	−131.0 (4)	C39—C40—C41—C42	0.3 (8)
N5—Ce—O6—C23	72.1 (4)	C40—C41—C42—C43	−1.0 (8)
N4—Ce—O6—C23	137.2 (4)	C40—C41—C42—C45	179.3 (5)
O2—Ce—O6—C23	−66.6 (4)	C41—C42—C43—C44	1.4 (8)
O4—Ce—O6—C23	−56.1 (4)	C45—C42—C43—C44	−178.9 (5)
Ce—O1—C2—C1	−154.3 (3)	C40—C39—C44—C43	0.3 (7)
Ce—O1—C2—C3	25.6 (5)	N2—C39—C44—C43	177.4 (4)
C7—C1—C2—O1	2.6 (7)	C42—C43—C44—C39	−1.0 (7)
C6—C1—C2—O1	−178.6 (4)	Ce—N4—C46—S1	−141 (8)
C7—C1—C2—C3	−177.3 (4)	Ce—N5—C47—S2	−10 (53)
C6—C1—C2—C3	1.5 (6)	Ce—N6—C48—S3	50 (19)

### Hydrogen-bond geometry (Å, °)

**Table:**

*D*—H···*A*	*D*—H	H···*A*	*D*···*A*	*D*—H···*A*
N1—H1A···O1	0.86	1.85	2.556 (4)	138
N2—H2A···O3	0.86	1.89	2.583 (4)	137
N3—H3A···O5	0.86	1.88	2.579 (4)	137
